# Is the First-Trimester Systemic Immune-Inflammation Index Associated With Preeclampsia?

**DOI:** 10.7759/cureus.44063

**Published:** 2023-08-24

**Authors:** Munire Funda Cevher Akdulum, Erhan Demirdağ, Seçil İrem Arık, Sahila Safarova, Mehmet Erdem, Nuray Bozkurt, Ahmet Erdem

**Affiliations:** 1 Obstetrics and Gynecology, Gazi University, Ankara, TUR

**Keywords:** maternal and fetal outcome, gestational hypertension, pregnancy, preeclampsia, systemic immune-inflammation index

## Abstract

Objective: Preeclampsia (PE) is a serious and common pregnancy issue. There is a systemic inflammation in PE and it is accompanied by increased oxidative stress, but the clear etiology has not been revealed. We aimed to predict PE with the systemic immune-inflammation index (SII) value calculated in the first trimester.

Material and methods: This is a retrospective study. One hundred fifty-seven pregnant women were included in the study. Twenty-seven pregnant women were excluded from the study. Age, gravida, parity, and hemogram values were recorded in the patients' first visit file records. The time and mode of delivery, birth weight, and APGAR scores were obtained from the file records of the patients. SII was created using the formula (neutrophil x platelet/lymphocyte).

Result: The study group included 30 pregnant women who had been diagnosed with PE. The control group consisted of the remaining 100 pregnant women. There was a statistically significant difference between PE and control groups in terms of SII (p=0.03). The SII level cut-off value for predicting PE was determined to be 836.83. This value's area was found to be 0.635 (0.519-0.752). Furthermore, the selectivity is 0.60 and the sensitivity is 0.40 for these values.

Conclusion: SII was found to be significantly higher in people with PE in the study. We showed that the SII value measured in the first trimester can be used to predict PE. It might make sense to combine this marker with the patient's history and other risk factors due to its low selectivity and sensitivity.

## Introduction

Preeclampsia (PE) is a serious and common pregnancy issue, which affects 2%-8% of pregnancies. It could be a sign of a severe maternal or fetal complication [1]. PE is diagnosed after the 20th gestational week when the arterial blood pressure measurement is 140/90 mmHg and above, accompanied by proteinuria or accompanied by thrombocytopenia, pulmonary edema, renal failure, impaired liver functions, and visual disturbances, according to the American College of Obstetricians and Gynecologists [2]. It is critical to identify pregnant women who are at high risk of developing PE or severe PE as soon as possible to avoid negative pregnancy outcomes. There is systemic inflammation in PE, and it is accompanied by increased oxidative stress, but the clear etiology has not been revealed [3]. Until now, precise PE estimation strategies have remained elusive. Like this, laboratory markers such as hematological parameters that can aid in the predictive evaluation of PE are worth investigating.

The neutrophil-to-lymphocyte ratio (NLR) is a systemic inflammatory marker [4]. If neutrophils and lymphocytes are exposed to excessively oxidized lipids in the placenta, they become overactive and secrete inflammatory cytokines, causing endothelial dysfunction [5]. The relationship between PE and NLR has been studied in many studies. There are conflicting results in this regard [6-8]. It has been reported that platelet and leukocyte activation in PE is higher than in healthy pregnancies [9]. Accordingly, platelet-to-lymphocyte ratio (PLR) was previously studied as an inflammatory marker to predict PE and its severity, but controversial results were found [10,11]. Systemic immune inflammation indices derived from peripheral blood cells have been studied recently because of their ease of measurement and computability [12].

The systemic immune-inflammation index (SII) is an inflammation marker based on neutrophil, platelet, and lymphocyte counts. It has been shown that SII has prognostic importance in various cancers and cardiac diseases [12-16]. While the relationship between SII and hypertension is a topic that is currently being discussed, PE and SII have not been studied yet. Early detection of PE allows for close clinical follow-up of patients and, with effective management, can safely prolong pregnancy. Our aim in this study was to investigate the value of SII as an indicator for PE.

## Materials and methods

This retrospective cohort study was conducted between February 2021 and February 2022. The study was approved by the Ethical Committee of the Institute (2022-889).

The study group (n=30) consisted of pregnant women diagnosed with PE. The control group (n=100) were pregnant women who gave birth without a diagnosis of PE. All patients in the study and control groups were cesarean deliveries.

Among the exclusion criteria of the study, there were multiple pregnancies and the mother had additional diseases such as diabetes mellitus, hypertension, kidney diseases, hematological diseases, and connective tissue diseases. Age, gravida, parity, and complete blood count values were recorded in the patients' first visit file records. As hemogram values in the first trimester, hemoglobin, platelet, neutrophil, and lymphocyte counts were analyzed. The time and mode of delivery, birth weight, and APGAR scores (first and fifth minutes) were evaluated from the file records of the patients.

The American Congress of Obstetricians and Gynecologists (ACOG) criteria were used to confirm a diagnosis of PE. PE is diagnosed after the 20th gestational week when the arterial blood pressure measurement is 140/90 mmHg and above, accompanied with or without proteinuria or accompanied by thrombocytopenia, pulmonary edema, renal failure, impaired liver functions, and visual disturbances.

The SII values were calculated from the hemogram values of the patients at the first visit in the first trimester. SII was created using the formula (neutrophil x platelet/lymphocyte) [12].

The IBM SPSS Statistics 22 program (IBM Corp., Armonk, NY) was used to analyze the data. When analyzing the study data, descriptive statistics (mean, standard deviation) for numerical variables are provided. The independent sample t-test and the chi-square test were used to compare the two groups. To investigate the relationship between numerical variables, Pearson correlation analysis was used. Furthermore, a SII cut-off value that could predict PE was investigated, and P 0.05 was accepted for significance.

## Results

One hundred fifty-seven pregnant women were included in the study. Twenty-seven pregnant women were excluded from the study. The study group included 30 pregnant women who were diagnosed with PE. The control group was formed from 100 pregnant women who were not diagnosed with PE.

While the age of women with PE was 31.5 ± 5.6 years, the mean age of the control group was 32.7 ± 5.3 years (p>0.05). In terms of age, gravida, and parity, there was no statistical difference between the groups (p>0.05). The difference in birth weights and delivery time was statistically significant (p=0.00). There was no statistical difference between the first and fifth-minute APGAR scores (p>0.05). Table 1 shows the comparison of demographic parameters and birth characteristics.

**Table 1:** Comparison of demographic features and birth characteristics *Data are given as mean ± SD; ¶ Data are given as median (minimum-maximum) (p < 0.05 was considered significant)

	Preeclampsia group (n=30)	Control group (n=100)	P-value
Age*	31.5 ± 5.6	32.7 ± 5.3	0.252
Gravida(median(minimum-maksimum) ¶	1.6(1-7)	1.8(1-8)	0.591
Birth weights(gr.)*	2365± 898	3086± 765	0.002
Birth time (week)*	34.4± 3.4	37.6± 2.1	0.002
APGAR 1. minute*	7.8±1.7	8.0±2.2	0.691
APGAR 5. minute*	9.3±1.3	9.2±2.0	0.634

While hemoglobin levels were 11.6 ± 1.4 g/dL in the study group, it was 11.9 ± 1.2 g/dL in the control group (p>0.05). The mean platelet count (10^9^/L) in the study group was 255.0 ± 82.5, while the control group had 243.7 ± 68.7 (p=0.45). Mean neutrophil counts (×10^9^/L) were in the study and the control groups were 7.81 ± 3.12 and 8.21 ± 3.40, respectively (p=0.56). Mean lymphocyte counts (×10^9^/L) were in the study and the control groups were 2.50 ± 0.98 and 2.11 ± 0.93, respectively (p=0.24). There was a statistically significant difference between PE and control groups in terms of SII (p=0.03) (Table 2).

**Table 2:** Difference between PE and control groups in terms of SII *Data are given as mean ± SD (p < 0.05 was considered significant) PE - preeclampsia

	Preeclampsia group (n=30)	Control group (n=100)	P-value
Hemoglobin(g/dL)*	11.6 ± 1.4	11.9 ± 1.2	0.230
Mean platelet counts (×10^9^ /L) *	255.0 ± 82.5	243.7 ± 68.7	0.451
Mean neutrophil counts (×10^9^/L)*	7.81 ± 3.12	8.21 ± 3.40	0.562
Mean lymphocyte counts (×10^9^/L)*	2.50 ± 0.98	2.11 ± 0.93	0.243
SII*	813.7 ± 394.1	1009.8 ± 590.4	0.031

**Figure 1:**
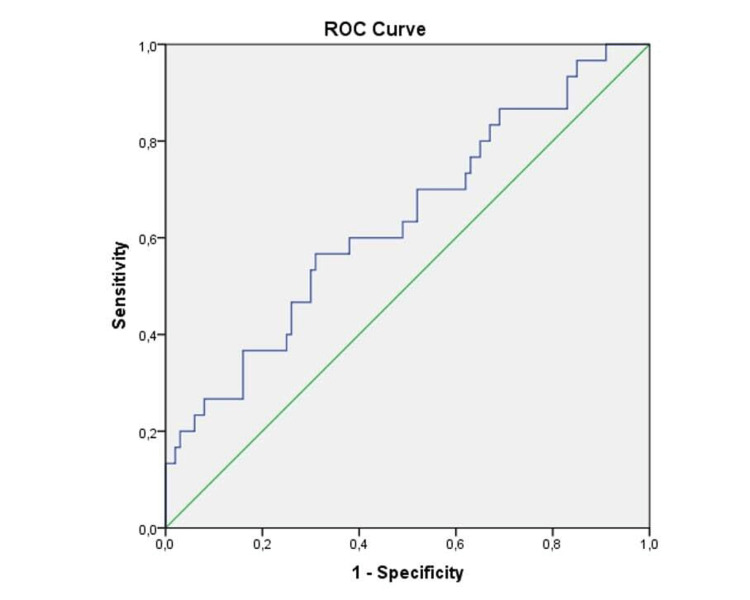
Area under ROC curve analysis of the systemic immune-inflammation index to predict preeclampsia

AUC and ROC were used to evaluate the performance of SII to predict PE. The SII level cut-off value for predicting PE was determined to be 836.83. This value's area was found to be 0.635 (0.519-0.752). Furthermore, the selectivity is 0.60 and the sensitivity is 0.40 for these values (Figure 1).

## Discussion

PE is a leading cause of maternal and neonatal mortality globally. Early PE prediction is an important aspect in the management of the disease and in improving pregnancy outcomes. There is no screening test to predict PE. SII was found to be significantly higher in people with PE in the study. We showed that the SII value measured in the first trimester can be used to predict PE. NLR and PLR, which were inflammation markers, have been studied before in the prediction of PE, but there was no study in the literature showing the relationship between SII and PE.

The relationship between hypertension and SII was investigated in a previous article [17]. SII was found to be significantly higher in patients with high intima thickness in the carotid artery. High SII has been associated with adverse outcomes of hypertension and negative outcomes in the endothelium [17]. There are studies showing that the immune system also plays a role in hypertension [18]. Angiotensin II is functional with lymphocytes. With the activation of lymphocytes, the damage of hypertension increases [18]. Inflammation markers, including lymphocytes, may be informative in terms of the severity and damage of hypertension [17]. Turgut et al. investigated whether SII could be used to predict miscarriages. High SII levels in early pregnancy have been linked to an increased risk of miscarriage. Abortions are performed for a variety of reasons. During this procedure, there is clearly an inflammatory activity in the uterus. According to this study, SII is also crucial in exhibiting the inflammatory response [19]. Tanacan et al. investigated the relationship between preterm premature rupture of membranes (PPROM) and SII in another investigation. In pregnant women with PPROM, an inflammatory reaction arises. SII was shown to be considerably greater in the PPROM group and more effective than NLR in detecting unfavorable pregnancy outcomes [20]. Orgul et al. examined the effect of magnesium sulfate treatment on maternal SII levels. Previous research had revealed that magnesium sulfate increases systemic inflammation through cytokines [21,22]. The injection of magnesium sulfate resulted in a considerable increase in SII in this study [23].

There have been numerous studies on the pathophysiology of PE, with one of the most well-known being: inflammatory stimulation causes an aberrant immunological response and produces arterial endothelial dysfunction, which leads to hypertension [24-27]. There was an inflammatory response and an increase in neutrophils in PE. With this increase, there was an increase in nitric oxide and superoxide radicals. These radicals cause endothelial damage and dysfunction [27-29].

The biggest limitation of the study is its retrospective design. The role of this index in predicting PE can be more clearly demonstrated by prospective studies with larger samples. 

## Conclusions

In this study, it was shown that there is a statistically significant relationship between PE and SII, which is an inflammatory marker. This finding shows that PE risk should be carefully considered when a high SII value is calculated in the first trimester. Due to its low selectivity and sensitivity, it may be reasonable to combine this marker with the patient's history and other risk factors. For the risk group, some drugs such as acetylsalicylic acid that can be used to prevent PE can be started early.
